# Water stable, red emitting, carbon nanoparticles stimulate 3D cell invasion *via* clathrin-mediated endocytic uptake†

**DOI:** 10.1039/d1na00813g

**Published:** 2022-01-26

**Authors:** Udisha Singh, Aditya Guduru Teja, Shanka Walia, Payal Vaswani, Sameer Dalvi, Dhiraj Bhatia

**Affiliations:** Biological Engineering Discipline, Indian Institute of Technology Gandhinagar Palaj Gujarat 382355 India dhiraj.bhatia@iitgn.ac.in; Chemical Engineering Discipline, Indian Institute of Technology Gandhinagar Palaj Gujarat 382355 India; Center for Biomedical Engineering, Indian Institute of Technology Gandhinagar Palaj Gujarat 382355 India

## Abstract

Bright fluorescent nanoparticles with excitation and emission towards the red end of the spectrum are highly desirable in the field of bioimaging. We present here a new class of organic carbon-based nanoparticles (CNPs) with a robust quantum yield and fluorescence towards the red region of the spectrum. Using organic substrates such as *para*-phenylenediamine (PPDA) dispersed in diphenyl ether under reflux conditions, we achieved scalable amounts of CNPs with an average size of 27 nm. These CNPs were readily taken up by different mammalian cells, and we show that they prefer clathrin-mediated endocytosis for their cellular entry route. Not only can these CNPs be specifically taken up by cells, but they also stimulate cellular processes such as cell invasion from 3D spheroid models. This new class of CNPs, which have sizes similar to those of proteinaceous ligands, hold immense potential for their surface functionalization. These could be explored as promising bioimaging agents for biomedical imaging and intracellular drug delivery.

## Abstract 2

Bright fluorescent nanoparticles with excitation and emission towards the red end of the spectrum are highly desirable in the field of bioimaging.

## Graphical abstract

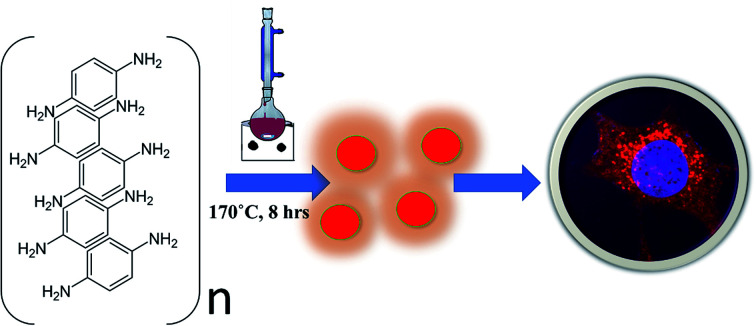

## 1 Introduction

Bioimaging of specific biomolecules, cellular processes, entire cells or tissues is one of the best non-invasive ways to understand and visualize biological activity at the cellular level.^1^ Recent years have witnessed the emergence of a plethora of fluorescent nanomaterials, having gained much attention in the field of bioimaging owing to their small sizes, low cost, highly tuneable fluorescent properties, and their ease of interfacing with biological systems leading to widespread applications.^2,3^ Many different fluorescent nanomaterials have already been used, such as rare-earth semiconductor quantum dots,^4,5^ organic dyes,^6^ and polymer dots.^7^

However, their applications are still extremely limited due to their limited stability, toxicity, and poor water stability, thus hindering their use for biomedical and biological applications. Carbon-based nanomaterials, especially carbon dots (CDs, including carbon nanoparticles, graphene quantum dots, and carbon quantum dots), have gained much attention because of their small size, biocompatibility, low toxicity, stable fluorescent properties, and low cost of synthesis.^8,9^ All these carbon nanomaterials have an sp^2^ hybridized carbon core and surface functional groups.^10^

Much research had already been done in the synthesis methods, doping and functionalization, tunable emission, *etc.* for CNPs. Still, it is challenging to tune the emission range of CNPs towards the red region of electromagnetic spectra. The structure of CNPs (by doping, surface chemistry, and size) and their synthesis methods significantly affect their emission properties. Most of the CNPs synthesised usually show fluorescence in the blue-green region, which is not of much use in bioimaging because their excitation is in the UV region, which not only induces autofluorescence to disturb the signal of CNPs but also damages the cells and tissues.^11,12^ CNPs emitting in the near-infrared region could overcome all the shortcomings of lower wavelength emissive CNPs and make them applicable for bioimaging purposes. Few studies on carbon nanoparticles showing fluorescence in the near-infrared region have been reported (ESI Table 1†). For example, Hui *et al.* synthesised red CDs with optical emission at 627 nm.^13^ Xiong and co-workers synthesised red emissive CDs with optical emission at 625 nm using PPDA and urea by the hydrothermal synthesis method.^14^ Although a few red emissive CNPs have been obtained showing fluorescence in the near-infrared region, the products obtained are in tiny amounts, and the synthesis process can be tedious, limiting the use of these CNPs. Also, the size and shape of nanoparticles matter a lot for their use in biomedical applications.

**Scheme 1:**
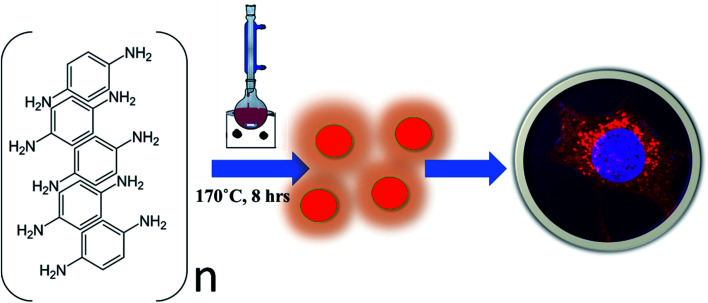
Schematic representation of CNP synthesis using *para*-phenylenediamine dissolved in diphenyl ether, refluxed at 170 °C for 8 h, leading to one-step formation of red emitting nanoparticles that could be taken up by cells for bioimaging applications.

We modified the existing methods in this direction by using *para*-phenylenediamine (PPDA) as the precursor material to achieve the synthesis of red emissive fluorescent CNPs. The CNPs obtained by reducing *para*-phenylenediamine (PPDA) exhibit excitation and emission spectra in the red region of the visible spectrum. The CNPs are synthesised by a one-step reflux reaction in a round bottom flask, which is simple and cost-effective, and a large amount of product is obtained with a yield of 87.65%, which is much higher as compared to other synthesis methods. To use the CNPs for biomedical applications, it is essential to know through which pathway they enter the cells. These CNPs were seen to be readily taken up by different mammalian cells *via* a very specific endocytic route called clathrin-mediated endocytosis (CME). The long-wavelength emission helps in avoiding the strong absorption from biological samples and preventing enhanced emission due to autofluorescence from the cells. Hua X. *et al.* also synthesised red CNPs with a size of approximately 2 nm using a similar precursor material *para*-phenylenediamine (PPDA). However, their quantum yield in water and ethanol compared to that of the CNPs we have synthesised is lower. They used hydrothermal methods of synthesis instead of the reflux condensation reaction we have used in the current study.^15^ Similarly, Wu and coworkers synthesised carbon dots with the highest emission at 530 nm wavelength and used them not only to stain the nucleolus but also for drug delivery and advanced bioimaging applications. They used *meta*-phenylene diamine instead of *para*-phenylenediamine as the precursor material and used the hydrothermal method of synthesis. They successfully transferred protoporphyrin IX into the nucleolus using carbon dots (CDs). The CNPs we have synthesised do not explicitly target any organelle, but they easily enter the cell cytoplasm and can be used for drug delivery, just like the CDs synthesised by Wu and coworkers.^16^ Not only were the CNPs actively taken up by cells, but they also stimulated different cellular physiological processes such as cell invasion in 3D spheroid models. Overall, our results indicate that the CNPs presented here are good bioimaging materials and can lay a path for the development of advanced bioimaging tools (Scheme 1).

## 2 Results and discussion

### 2.1 Synthesis and characterization of CNPs

**Fig. 1:**
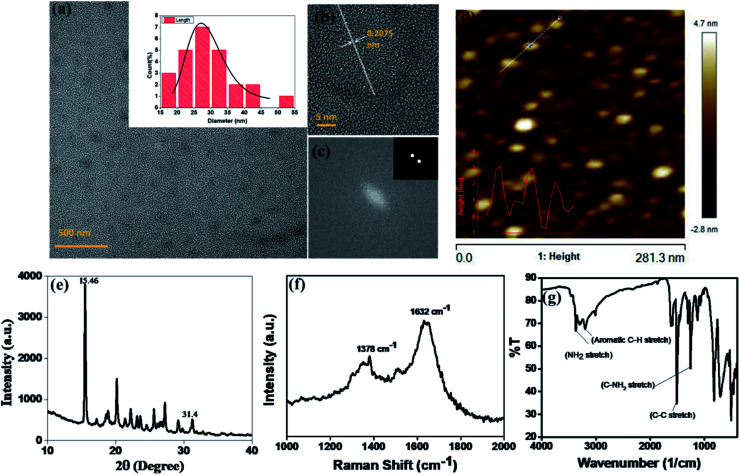
Characterization of fluorescent CNPs. (a) Transmission electron microscopy (TEM) image shows spherical-disk shaped CNPs of size 26.7 ± 3.2 nm. (b) High resolution transmission electron microscopy (HRTEM) image of CNPs showing lattice fringes of 0.2075 nm. (c) Fast Fourier transform image of CNPs exhibiting (1 0 1) lattice planes. (d) Atomic force microscopy (AFM) image of CNPs showing the similar sized CNPs with a topological height of 2.6 nm. (e) X-Ray diffraction (XRD) spectra of CNPs showing that CNPs are crystalline. (f) Raman spectra of CNPs giving D and G band values of 1378 cm^−1^ and 1632 cm^−1^ respectively. (g) Fourier transform infrared (FTIR) spectra of CNPs showing the functional groups present on the surface of CNPs.

Red fluorescence emitting CNPs were synthesised through reflux reaction mediated pyrolysis of *para*-phenylenediamine (PPDA) in diphenyl ether for 8 h at 170 °C. The size and morphology of the CNPs were characterized using transmission electron microscopy (TEM). TEM images show that these particles are adequately dispersed and possess a spherical disk-like structure. The mean diameter of the fluorescent CNPs is 26.7 ± 3.256 nm while counting 100 nanoparticles displayed in Fig. 1a. Particle size measurement was performed with ImageJ software using a scaled image and analysed using Origin software. From the high-resolution transmission electron microscopy (HRTEM) image in Fig. 1b and c, lattice fringes with a lattice distance of 0.2075 nm, which is in good accordance with the (101) carbon planes, were obtained, suggesting that the CNPs synthesised are crystalline in nature.^17^ The atomic force microscopy (AFM) image reveals a topological height of 2.6 nm, indicating that fluorescent CNPs contain an average of 7 to 8 layers of graphene sheets (Fig. 1d).^18^

To further understand the structure and composition of CNPs, we performed X-ray diffraction spectroscopy (XRD), and Raman and Fourier transform infrared (FT-IR) spectroscopies of fluorescent CNPs. The XRD profile of CNPs shows several peaks that may be due to the ordered stacking of the synthesised CNPs. The number of peaks also shows the complex mixture of crystalline components in the sample solution of CNPs.^19^ The peaks at 15.46° and 31.4° correspond to the precursor material PPDA, revealing some of the precursor material's chemical structure is still intact even when the CNPs are formed as shown in Fig. 1e. Raman spectroscopy was also used as a non-destructive way to understand the degree of graphitization of CNPs. We obtained two peaks at 1378 cm^−1^ and 1632 cm^−1^ representing the D band and G band, respectively (Fig. 1f). The D band constitutes the vibrations of disordered graphite or glassy carbon. The G band represents the in-plane displacement of carbon atoms in a two-dimensional hexagonal lattice. Also, the value of *I*_D_/*I*_G_ is being used to evaluate the degree of graphitization.^21^ The intensity of the G band is higher than the peak of the D band with a *I*_D_/*I*_G_ value of 0.844 (Fig. 1f), implying that more sp^2^-hybridized carbon is present compared to sp^3^ hybridized carbon. The FTIR spectrum gives a qualitative idea about the functional groups present on the surface of CNPs. CNPs mainly contain C, O and N elements forming chemical bonds –NH_2_ (3364 cm^−1^), C–C (1510 cm^−1^) and C–NH_2_ (1257 cm^−1^) as shown in Fig. 1g. The absorption peak obtained at 3364 cm^−1^ confirms the presence of an amine group on the surface of FCNPs, also proving the nitrogen doping of CNPs. The C–H stretching occurs around 3180 cm, ensuring that the CDs contain the aromatic ring of carbon.

### 2.2 Optical properties of CNPs reveal stable, pH-responsive, red-emitting fluorescence

**Fig. 2:**
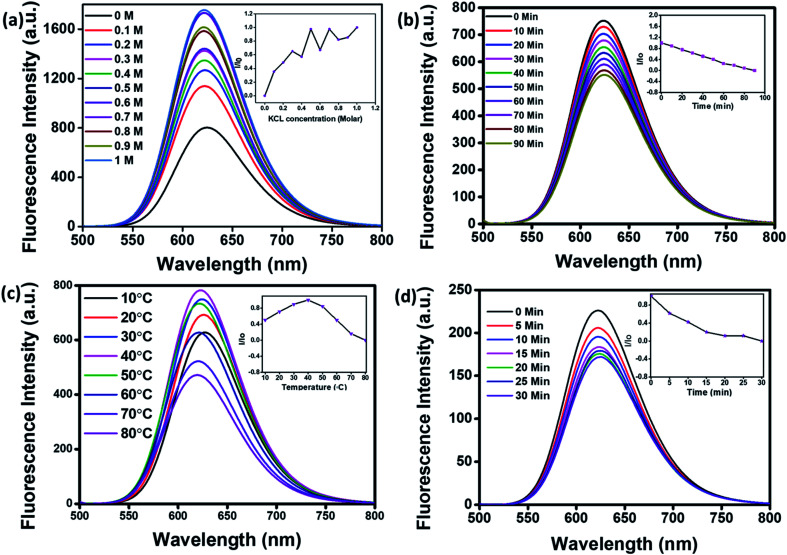
(a) Ultraviolet (UV)-visible spectra of CNPs showing three absorbance peaks at 241 nm, 306 nm and 415 nm corresponding to the π–π* transition from the aromatic carbon structure, π–n* transition from functional groups with lone pair electrons and the energy level transition from the angstrom-sized conjugated π-structure present in CNPs. (b) Excitation and emission spectra of CNPs at 480 nm excitation wavelength showed *λ*_max_ at 622 nm wavelength. (c) Emission spectra of CNPs at different excitation wavelengths ranging from 400–520 nm with emission at a *λ*_max_ of 622 nm with varying intensities. (d) Emission spectra of CNPs dispersed in different pH solutions (3, 5, 7 and 12).

To understand the optical properties of CNPs, UV-visible absorption and fluorescence emission and excitation spectra were measured. We obtain three absorbance peaks in the UV-vis spectra at 241 nm, 306 nm, and 415 nm (Fig. 2a). The absorbance peak at 241 nm is assigned to the π–π* transition from the aromatic carbon structure, the shoulder peak at 306 nm is assigned to the π–n* transition from functional groups with lone pair electrons and the absorbance peak at the longer wavelength 415 nm is assigned to the energy level transition from the angstrom-sized conjugated π-structure present in CNPs.^22^ The fluorescence emission spectra of fluorescent CNPs were measured as a function of different excitation wavelengths. The fluorescence (FL) spectra of CNPs show a maximum peak at 622 nm with an excitation wavelength of 480 nm. As shown in (Fig. 2b), the FL spectra of CNPs don't shift with respect to the change in the excitation wavelength of 400 to 520 nm range, indicating that fluorescent CNPs have distinct excitation independent FL behaviour, which is very seldom observed. This also shows that the CNPs exhibit a quite homogeneous size distribution, composition and surface state. The sp^2^ hybridized carbon network in CNPs can also be one of the reasons for independent excitation fluorescence. The CNPs show a brownish-red colour under white light and a dark red colour under UV light illumination in aqueous solution, as shown in the inset of Fig. 2a.

The fluorescence quantum yield of optimal CNPs is 5.86% in Milli-Q water and 87% in ethanol by referring to the standard rhodamine B (QY = 33%). This also shows that the synthesised CNPs are more dispersed in organic solvents and show a higher QY and fluorescence. The CNPs synthesised were redispersed in different solvents: water, hexane, acetone, acetonitrile, dimethyl sulfoxide (DMSO), *N*,*N*-dimethyl formamide (DMF), ethyl acetate, isopropyl alcohol (IPA), ethanol and methanol. All ten solvents have different polarities, and their emission spectra also shift with a change in solvent, as shown in ESI Fig. S2a.† The colour of the CNPs changes in different solvents showing that the fluorescence of CNPs not only depends on functional groups present on their surface but also the type of the dispersant used.^20^

For cellular stability applications, pH-dependent studies of CNPs were done by monitoring changes in their fluorescence signal as a function of pH. Different pH solutions in Milli-Q water were prepared (3, 5, 7 & 12) and CNPs (0.5 mg mL^−1^) were dispersed in it, and emission spectra were recorded as shown in Fig. 2d. The fluorescent CNPs show an increase in fluorescence with an increase in pH, hence showing great potential in pH sensing. The lowest fluorescence intensity was obtained at pH 3 and the highest intensity at pH 12. The percentage increase in intensity from pH 3 to pH 7 is 38%, whereas the increase in fluorescence intensity from pH 7 to pH 12 is approximately 31%. The pH sensitivity of CNPs may be due to protonation and deprotonation of oxygen-containing groups on the surface.^23^ Also, there is a significant colorimetric change of CNPs dispersed in different pH solutions.

**Fig. 3:**
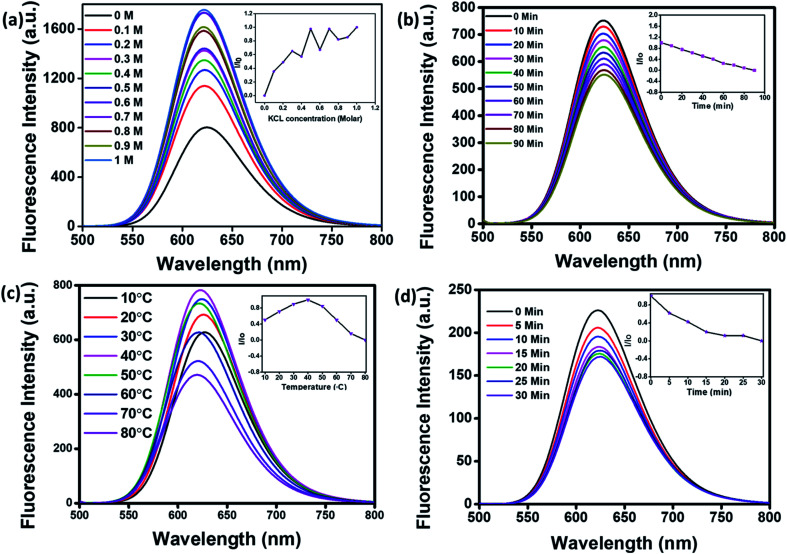
Photostability studies of CNPs. (a) Fluorescence emission spectra of CNPs titrated with different ionic concentrations of potassium chloride (KCL) (0 to 1 M). (b) Fluorescence spectra of CNPs recorded from 0 to 90 min at increments of 10 min, irradiated with an excitation wavelength of 480 nm continuously to check their photostability. (c) Fluorescence spectra of CNPs at different temperatures (10–80 °C) recorded at increments of 10 °C. (d) Fluorescence spectra of CNPs at different time points (0–30 min) when kept at 80 °C. The inset shows the relative effect on the photostability of CNPs excited under various conditions.

For application in cellular bioimaging, the stability of fluorescent CNPs in different ionic concentrations is very crucial. To check the fluorescence stability of CNPs in a highly ionic environment, we dispersed CNPs (0.5 mg mL^−1^) in different concentrations of potassium chloride (KCl) salt (0 to 1 M) solution. Our results show a significant increase in the fluorescence of CNPs with an increase in the concentration of KCl (Fig. 3a). We also analysed the photostability of CNPs under constant visible light at 480 nm. The CNPs were irradiated with uninterrupted excitation wavelength light for 90 min, and readings were taken at an interval of every 10 min. The resultant fluorescence intensity (*I*/*I*_o_) stipulates an insignificant decrease in fluorescence with time, indicating the photostability of synthesized CNPs (Fig. 3b).

We further studied the thermal stability of CNPs using a fluorescence spectrophotometer and changed the temperature from 10 °C to 80 °C. With increase in temperature, initially, the fluorescence intensity increased from 10 °C to 40 °C followed by a decrease from 50 °C to 80 °C (Fig. 3c). The increase in fluorescence intensity from 10 °C to 40 °C is 20%, and the reduction in fluorescence intensity from 50 °C to 80 °C is 40%, indicating that the fluorescence intensity of CNPs is temperature-dependent, and the best fluorescence is obtained at physiological temperatures of cells and tissues. The CNPs were also kept at 80 °C for 30 minutes to check their thermal stability. There was an insignificant decrease in the fluorescence intensity of CNPs with time at 80 °C (Fig. 3d). The reduction in fluorescence intensity with increase in temperature from 40 °C to 80 °C can be explained by thermally activated non-radiative channels. At high temperatures, the non-radiative channels are activated, leading to excited electrons not effectively releasing photons. This is why we see a reduction in PL intensity at high temperatures.^24^

### 2.3 Cellular uptake of CNPs follows clathrin-mediated endocytosis

To check the suitability of the developed CNPs as molecular imaging probes toward biological systems and also to monitor the effect of the same on normal and cancer cells, microculture tetrazolium (MTT) assay was performed with mesenchymal triple-negative breast cancer cells (SUM159A) and mouse embryonic fibroblast (MEF) cells (ESI Fig. S5a and b).† The experiments were carried out using different concentrations, namely 10, 20, 30, 50, 100, 200, and 500 μg mL^−1^ of CNPs. After 24 h of incubation with CNPs, SUM159A showed ∼80% cell viability up to 50 μg mL^−1^ concentration, decreased to ∼62% up to 200 μg mL^−1^ and finally 52% at 500 μg mL^−1^ of CNP concentration (ESI Fig. SI5a†). In the case of MEF, the CNPs showed cell viabilities of 70% and 50% at a concentration of 10–20 μg mL^−1^ and 30–100 μg mL^−1^, respectively. The cell viability percentage significantly dropped, with ∼37% and 16% cell viabilities at 200 and 500 μg mL^−1^ concentrations of CNPs. The cytocompatibility studies with SUM159A suggested that CNPs show high toxicity at a concentration of 100 μg mL^−1^ even after 24 h, whereas the same limit for MEF was found to be 20 μg mL^−1^. This also suggests that different cells have different capacities to interact with CNPs, thus affecting their cytotoxicity.

**Fig. 4:**
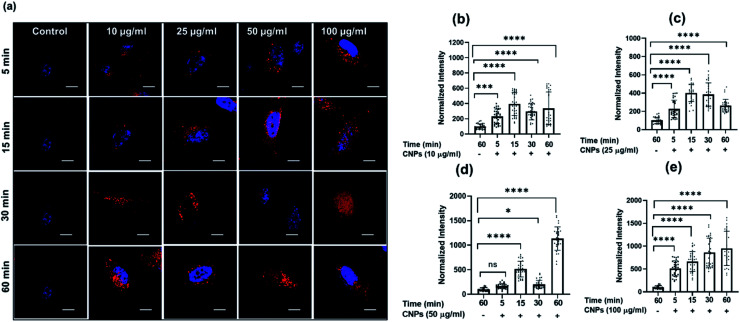
Concentration-dependent cellular uptake studies of CNPs at different time intervals. (a–d) Confocal images of SUM159A incubated with CNPs for 5, 15, 30, and 60 min, respectively. The scale bar is 10 μm for all the images. The first column in the figure represents untreated SUM-159A cells imaged after 60 min of incubation. (e) Quantification of cellular uptake of CNPs at 5, 15, 30, and 60 min, respectively. The scale bar is 5 μm. **** indicates a statistically significant value of *p* < 0.0001, *** indicates a statistically significant value of *p* = 0.0006, ** indicates a statistically significant value of *p* = 0.0074 and * = 0.03 (one-way ordinary ANOVA).

**Fig. 5:**
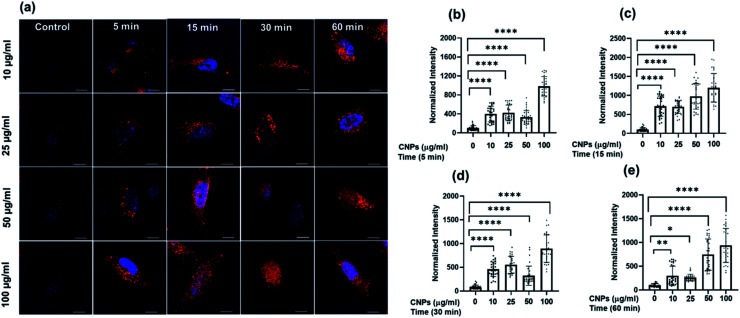
Time-dependent cellular uptake studies of CNPs at different time intervals. (a) Confocal images of SUM 159 incubated with 10, 25, 50, and 100 μg mL^−1^ of CNPs, respectively. The first column in the figure represents untreated SUM-159 cells imaged after 60 min of incubation. The scale bar is 10 μm for all the images. (b–e) Quantification of cellular uptake of CNPs at 10, 25, 50, & 100 μg mL^−1^, respectively. The scale bar is 5 μm. **** indicates a statistically significant value of *p* < 0.0001, ** indicates a statistically significant value of *p* = 0.002. and * = 0.01 (one-way ordinary ANOVA).

To explore the cellular uptake properties of CNPs, confocal microscopy studies were done to analyse the time and concentration-dependent cellular internalization of CNPs using SUM159A and MEF cells (Fig. 4 and SI4i,† respectively). Cells, when incubated with CNPs of different concentrations at 37 °C and analysed by using a confocal microscope, revealed the successful internalization of CNPs. Further, the confocal images and quantification data suggested that CNPs showed a strong intracellular fluorescence response regarding time and concentration. Concentration-dependent studies showed that the fluorescence intensity was increased ∼1–2 times from 10 to 100 μg mL^−1^ concentration of CNPs. The cell morphology was retained unchanged at lower concentrations. However, when cells were exposed to a higher concentration of 100 μg mL^−1^ CNPs, the fluorescence signal was observed from the nucleus, and cells appeared to be in stress (Fig. 4a and ESI Fig. SI6i).† Further, to evaluate the time dependent uptake of CNPs, the same data from Fig. 4 were plotted with respect to different time intervals. The time-dependent studies showed an increase in fluorescence signal up to 60 min with enhanced fluorescence intensity (Fig. 5 and ESI Fig. SI6ii).† Confocal microscopy studies showed that the synthesized CNPs could be used for bioimaging applications due to their stable and robust fluorescence emission in the red channel.

**Fig. 6:**
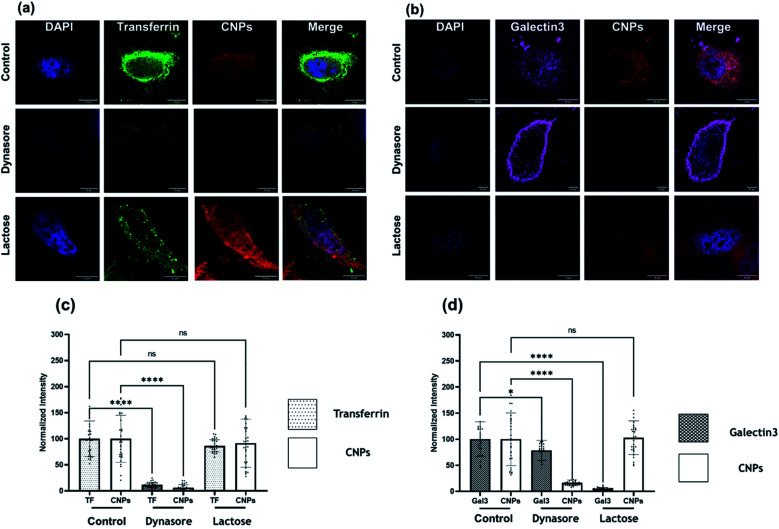
Uptake of CNPs *via* the endocytosis pathway in SUM-159 cells under conditions that perturb CME and CIE. (a) Uptake of CNPs and A488-labelled transferrin (Tf) in cells treated with dynasore (80 μM) and lactose (150 mM). (b) Quantification of normalized intensity. (c) Uptake of CNPs and A647-labelled galectin-3 (Gal3) in cells treated with dynasore (80 μM) and lactose (150 mM). The scale bar is 10 μm for all the images. (d) Quantification of normalized intensity. **** indicates a statistically significant value of *p* < 0.0001. * Indicates a statistically significant value of *p* = 0.376 and ns indicates a non-significant value of *p* (one-way ordinary ANOVA).

Clathrin-mediated endocytosis (CME) and clathrin-independent endocytosis (CIE) are the most critical pathways considered for NP uptake.^25^ We thus investigated the plausible endocytosis pathway responsible for the cellular uptake of CNPs by using cellular cargo markers *viz.*, transferrin (Tf), a well-known target of CME, whereas galectin3 (Gal3) for marking CIE was included as a reference. Specific inhibitors, namely dynasore to block CME and lactose for CIE inhibition, were used to validate their effect on CNP uptake. When cells were pulsed with these markers and CNPs in the presence of these inhibitors, we observed that Tf and CNP uptake were significantly reduced by dynasore treatment in SUM-159A cells (Fig. 6a and b). At the same time, lactose inhibition resulted in a non-significant change in fluorescence intensity in the case of Tf and CNPs, while the intensity of Gal3 uptake was significantly decreased.

We also examined the cellular uptake of CNPs *via* the CIE pathway using Gal3 as a reference marker. Lactose is known to compete with extracellular Gal3 for binding to specific β-galactosides on the plasma membrane receptors and, therefore, used to inhibit the uptake of extracellular Gal3.^26^ It was observed that the uptake of Gal3 was significantly decreased when inhibited by lactose (CIE blocker), whereas the CNP uptake was marginally affected in cells (Fig. 6c and d). However, upon inhibition *via* dynasore, the uptake of Gal3 was only marginally reduced. On the contrary to this, CNP uptake was decreased significantly upon treatment with dynasore, indicating the strong involvement of dynamin which plays the role of scission of the clathrin-coated vesicles formed on the plasma membrane. These results confirmed our previous observation that CNP uptake was CME dependent.

### 2.4 CNP uptake stimulates cellular invasion in 3D spheroid models

**Fig. 7:**
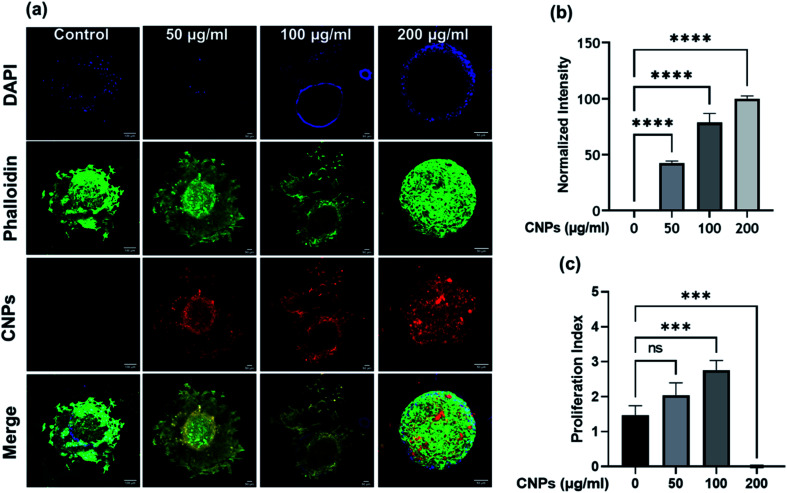
(a) Confocal microscopy images of 3D spheroids treated with 50, 100, and 200 μg mL^−1^ of CNPs for 24 h. The scale bar is 100 μm in the control sample and 50 μm for the rest of the images. The scale bar is 100 μm for merged images and 50 μm for all other channels. (b) Quantification data of normalized intensity of CNPs. (c) Quantification data of proliferation index. **** indicates a statistically significant value of *p* < 0.0001, *** indicates a statistically significant value of *p* < 0.001, whereas ns indicates non-significant data (one-way ordinary ANOVA). *N* = 3 spheroids per condition.

Three-dimensional (3D) spheroid-based cancer models are well explored to study tumour progression, cell activity, and the effect of pharmacokinetic drugs. Three-dimensional spheroids provide better insight into the disease state and proliferation, invasion, or migration of tumour cells. Therefore, we further explored the cellular uptake of CNPs by 3D spheroids and its effect on cell invasion in a 3D matrix. Spheroids were made from triple-negative breast cancer MDA-MB-231 cells using the hanging drop method. A T25 flask having approximately 85% cellular confluency was used for preparation of spheroids. We use 1 mL trypsin for trypsinization and add 4 mL DMEM complete media. Media containing the cells were placed on a Petri dish lid in the form of droplets and put upside down, covering the Petri dish, which was filled with around 30 mL PBS. PBS gives a moist environment for spheroid growth. The excellent quality spheroids are collected from the hanging drop by softly dispersing in 50 μL of collagen-complete media (at 2 : 1 vol ratio). These spheroids were suspended in collagen with or without CNPs. The spheroids were allowed to grow in the matrix for 24 h at 37 °C, after which they were fixed using paraformaldehyde (4% PFA), stained for nucleus using DAPI and actin-cytoskeleton using Phalloidin green.^27^ Spheroids treated with three different concentrations of CNPs *viz.*, 50, 100, and 200 μg mL^−1^ were incubated for 24 h at 37 °C (Fig. 7a and b). After staining and fixing, the spheroids were mounted on glass slides using Mowiol as a mounting medium. The prepared glass slides were then used for analysis on a Leica DMI8 Confocal microscope. 10× magnification is used for imaging of spheroids treated with different concentrations of CNPs. Images were in the form of *z*-stacks. Three lasers of 405 nm, 488 nm and 561 nm were used as excitation sources for confocal microscope imaging. Cells, while migrating in 3D, had actively endocytosed the CNPs from the collagen matrix, probably *via* the CME pathway. To study the effect of CNPs on the invasion capacity of cells in 3D spheroid models, the invasion index was plotted. The cell invasion index of every 3D tumour cell was calculated using the formula‬‬‬‬‬‬‬‬‬‬‬‬‬‬‬‬‬‬‬‬‬‬‬‬‬‬‬‬:Cell invasion index = distance migrated by the cells from the surface of spheroids/diameter of core spheroid

The migration distances were calculated using ImageJ software. To study the effect of CNPs on the invasion capacity of cells in 3D spheroid models, the invasion index was plotted, which showed that 3D tumour models treated with 100 μg mL^−1^ of CNPs had a cell invasion index of approximately 3, which was higher than the cell invasion index obtained for the tumour model for the 50 μg mL^−1^ and control samples (Fig. 7c). In the 200 μg mL^−1^ sample, there was no proliferation observed, indicating an inhibitory effect of CNPs on cell invasion at higher concentrations.

## 3 Conclusions

We designed near-infrared red emissive CNPs by the reflux reaction method of heating using *para*-phenylenediamine (PPDA) as the precursor material and diphenyl ether as the solvent. Owing to red emissive fluorescence and good biocompatibility, the CNPs have great potential in the field of bioimaging.^28–32^ Different characterization techniques were used to properly analyse the structure, morphology, and optical properties of CNPs. The QY of CNPs is ∼6% in water and 87% in ethanol, showing that the fluorescence of CNPs also depends on the type of solvent used. The fluorescence intensity of CNPs also depends on the pH of the dispersing medium (water), which increases with an increase in pH. Ionic stability, photostability, and thermal stability were also checked. In cell viability studies, we observed that CNPs did not show toxicity up to 100 μg mL^−1^ even after 24 h in SUM159A cells. Concentration-dependent and time-dependent studies on cellular uptake of CNPs were done and observed that most CNPs were taken up into the cells through clathrin-mediated endocytosis. The same effect was observed not only in 2D but also in the 3D spheroid model, and it was observed that CNPs could trigger the invasion of MDA-MB-231 cells in a collagen matrix. The present work provides not only a promising fluorescent nanomaterial for bioimaging but also opens other platforms for modulating the surface properties of nanoparticles along with the capacity to bioconjugate to different biomolecules, which will enable widespread biological and biomedical applications of CNPs in the future.

## 4 Materials and methods

### 4.1 Materials

*Para*-phenylenediamine (PPDA), Moviol, Hoechst, dynasore, galectin3 (Gal3) (alexa 647), transferrin (Tf)-A488, and 3-(4,5-dimethylthiazol-2-yl)-2,5-diphenyltetrazolium bromide (MTT) were obtained from Sigma-Aldrich. Diphenyl ether (99%) was obtained from Avra, and *n*-hexane of HPLC grade, acetone (>99.5%), *N*,*N*-dimethyl formamide (>99.5%), ethanol(>99.5%), methanol (>99.8%), acetone, acetonitrile, isopropyl alcohol (IPA) and lactose were purchased from Merck. Paraformaldehyde, dimethyl sulfoxide (DMSO), rhodamine B, and cell culture dishes for adherent cells (treated surface) were procured from Himedia. Ham's Nutrient Mixture F12 (HAMs F12), Dulbecco's modified Eagle's medium (DMEM), fetal bovine serum (FBS), penicillin–streptomycin, trypsin–EDTA (0.25%), and collagen1 rat tail were purchased from Gibco. All the chemicals were of analytical grade and no further purification was required.

### 4.2 Synthesis of fluorescent CNPs

Highly fluorescent red light-emitting CNPs were synthesised using the pyrolysis method by a reflux reaction.^19^ We have modified the experimental conditions to obtain the CNPs of desired size and properties. *Para*-phenylenediamine (PPDA) is used as the precursor material and dissolved in diphenyl ether. The reaction takes place for eight hours at 170 °C. After the heating process, the product was allowed to cool down to room temperature. The CNPs synthesised were precipitated out using hexane by the solvent displacement method. The product obtained after heating was added dropwise to 100 mL of hexane solution for the separation of CNPs. The precipitated CNPs were centrifuged and washed three times using hexane and then kept under normal atmospheric conditions to dry up.

### 4.3 Various analytical methods used for the characterization of CNPs

The shape and size of the fluorescent CNPs were analysed using an FEI Titan Themis transmission electron microscope (TEM) (60–300 kV). An atomic force microscope (AFM) was used to obtain both two-dimensional (2D) and three-dimensional (3D) images of CNPs. The X-ray diffraction (XRD) technique was used to characterize the crystalline nature of CNPs and for phase identification. The patterns from an X-ray diffractometer (Brüker-D8 Discover) were recorded with a speed of 0.2° min^−1^ from 10° to 60° with Cu K_α_ radiation. Raman spectroscopy was performed using a Kaiser Raman spectrometer with a 785 nm YAG laser. The fluorescence spectra were obtained using an FP-8300 Jasco spectrofluorometer (Japan) in the excitation range of 400 to 520 nm. Rhodamine B was used as a standard reference dye for fluorescence measurement, with a relative quantum yield of 5.86% in water and 87% in ethanol. UV-vis absorbance spectra of CNPs were obtained by using a Spectrocord-210 Plus Analytikjena (Germany). Fourier transform infrared spectroscopy (FT-IR) spectra of CNPs were recorded by using an FTIR spectrometer from Spectrum 2, PerkinElmer in the ATR mode. Scanning was done in the range of 400 cm^−1^ to 4000 cm^−1^. The cell imaging for the fixed samples was performed under 63× resolution using a confocal laser scanning microscopy platform Leica TCS SP8.

### 4.4 Spectroscopic studies

The solvents used to record absorption and emission spectra are of spectroscopic grade. The emission spectra were recorded 10 nm after the excitation wavelength for the standard recording of fluorescence spectra. Quantum yields were determined by comparison with rhodamine B in ethanol (*ϕ* = 0.33) as a reference using the following equation:
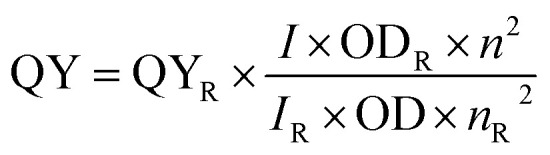


where QY is the quantum yield, *I* is the integrated fluorescence intensity, *n* is the refractive index, OD is the optical density of that particular excitation wavelength, and R is used for reference.

### 4.5 Cell culture

Mesenchymal triple-negative breast cancer cell lines (SUM159A) and embryonic mouse fibroblast (MEFs) cells were obtained as a gift from Prof. Ludger Johannes, Institut Curie, Paris, France. SUM159A and MEF cells were cultured in HAM’s F12 and DMEM supplemented with 10% FBS and 1% penicillin–streptomycin. For all the studies, PBS of 1× strength with pH 7.4 was used.

#### 4.5.1 MTT assay

To assess the toxicity of the CNPs, the cells were seeded in a 96-well plate with a cell density of 10 000 cells per well (100 μL) and incubated for 24 h in a CO_2_ incubator, maintained at 5% CO_2_ and 95% humidity, for cell adherence. After that, the cells were washed with PBS and treated with 100 μL of 4 different concentrations of CNPs *viz.*, 10, 20, 30, 50, 100, 200, & 500 μg mL^−1^, in triplicate and were incubated for 24 h. After the completion of incubation time, a colorimetric 3-(4,5-dimethylthiazol-2-yl)-2,5-diphenyltetrazolium bromide (MTT) assay was performed to quantify the effect of CNPs on cell viability. At the end of the treatment, cells were washed twice with PBS; then 10 μL of MTT solution (5 mg mL^−1^) was added to each well, and the solutions were further incubated for 3 h at 37 °C. After incubation, the formazan crystals formed were dissolved in 100 μL DMSO and incubated in the dark for 15 min. The intensity of the colour was then measured from the absorbance at 570 nm from the formazan crystals and was representative of the number of viable cells per well. The values thus obtained for the untreated control samples were equated to 100%, and the relative percentage values for CNPs were calculated accordingly. All experiments were performed in triplicate, and the cell viability (%) was calculated using eqn (1).1
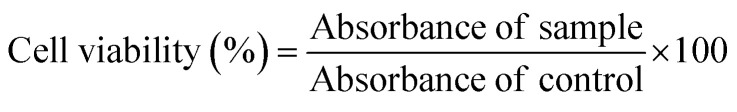


#### 4.5.2 Confocal microscopy studies

SUM 159 and MEF cells were seeded in HAMS F12 and DMEM at a density of ∼40 000 cells per well on coverslips in 24 well plate cell culture plates and incubated for 24 h at 37 °C and 5% CO_2_. After incubation, the cells were washed twice with PBS and treated with 10, 25, 50, and 100 μg mL^−1^ of CNPs, and all the samples were further incubated for different time points of 5, 15, 30, and 60 min, respectively. After desired incubation time, the cells were washed twice with PBS and then fixed using 4% PFA for 20 min. Afterwards, the cells were washed with PBS thrice, stained with DAPI, and mounted using a mounting medium (moviol). Finally, cellular internalization of CNPs was examined through Leica TCS SP5 confocal microscope with a 63× oil immersion objective. A 405 nm laser was used as the excitation source for DAPI, while a 488 nm argon laser was used as an excitation source for CNPs. The emission bandwidth for DAPI and CNPs was 420–450 nm and 590–700 nm, respectively.

#### 4.5.3 Cellular uptake studies *via* endocytosis

We next wanted to explore the pathway by which CNPs entered the cells. Briefly, SUM-159 cells were seeded on coverslips placed in a 24 well plate with a cell density of 40 000 cells per well. The cells were treated with dynasore (80 μM) and lactose (150 mM) in HAM’s F-12 (serum-free media) in desired wells and incubated for a specific time interval at 37 °C. Dynasore and lactose were used to inhibit clathrin-mediated endocytosis (CME) and clathrin-independent endocytosis (CIE) pathways, respectively. The untreated cells were used as a control. Afterwards, the cells were washed gently and incubated with fluorescently labelled markers, which follow CME (Tf (5 μg mL^−1^)) and CIE (Gal3 (5 μg mL^−1^)) pathways and CNPs (25 μg mL^−1^) for a specific time interval at 37 °C. The cells were washed thrice with PBS and fixed using 4% PFA for 15 min at 37 °C. The coverslips were mounted on Moviol containing Hoechst for imaging. Microscopy imaging of the fixed cells was performed using a confocal laser scanning microscope under 63× resolution. Quantification of data was done using Fiji ImageJ software.

#### 4.5.4 Cell invasion assay using 3D spheroid models

3D spheroids were prepared by using the hanging drop method. A T25 flask with 85 percent confluency was trypsinized with 1 mL trypsin to harvest the cells from the culture flask. Two millilitres of a complete fresh medium were added to the trypsinized solution and thoroughly mixed to inactivate trypsin. The mixture containing cells was centrifuged at 500 g for 3 minutes. The pellet obtained was resuspended in 4 mL of the complete fresh medium and redispersed. Then 35 μL of medium-contained cells were applied as drops on the inside surface of the Petri dish cover. To provide a moist environment for spheroid formation, 15 mL of phosphate buffer saline was poured into the Petri dish. The cell droplet-containing Petri dish lid was turned upside down, covering the Petri dish, and placed for incubation at 37 °C in an incubator. 3D spheroids were formed after 36 h of incubation. Each drop consists of one spheroid. The spheroids collected from the hanging drop are further used for cell proliferation assay. Spheroids collected from the hanging drop were added to the ECM matrix containing collagen and media in 2 : 1 ratio, respectively. After the transfer to the matrix, the spheroids were subjected to the respective treatments with CNPs. After 24 h, the spheroids were fixed using 4% PFA and actin and nuclei were marked by using the phalloidin–alexa fluor 488 stain and Hoechst. The spheroids were mounted onto slides and further imaged using a Leica confocal microscope.

## Conflicts of interest

The authors declare no conflict of interest.

## Supplementary Material

NA-004-D1NA00813G-s001
