# Supplementary material for: Water stable, red emitting, carbon nanoparticles stimulate 3D cell invasion via clathrin-mediated endocytic uptake
Source: Nanoscale Adv. 2022 Jan 26;4(5):1375–86. doi: 10.1039/d1na00813g (PMC9418831; doi:10.1039/d1na00813g)
Supplement: NA-004-D1NA00813G-s001 [file NA-004-D1NA00813G-s001.pdf]

Electronic Supplementary Material (ESI) for Nanoscale Advances.
This journal is © The Royal Society of Chemistry 2022

Supplementary Information

Water stable, red emitting, carbon nanoparticles stimulate 3D cell invasion via clathrin-mediated endocytic uptake

Udisha Singh[1#], Aditya Teja[2#], Shanka Walia[1#], Payal Vaswani[1], Sameer Dalvi[2,3] and Dhiraj Bhatia[1,3]

[1]Biological Engineering Discipline, Indian Institute of Technology Gandhinagar, Palaj, Gujarat 382355, India

[2] Chemical Engineering Discipline, Indian Institute of Technology Gandhinagar, Palaj, Gujarat 382355, India

[3] Center for Biomedical Engineering, Indian Institute of Technology Gandhinagar, Palaj, Gujarat 382355, India

[#] US, AT, SW contributed equally

*Correspondence to – dhiraj.bhatia@iitgn.ac.in

SI table 1: Different red emissive carbon nanoparticles synthesised in recent years

No.	Reactants	Method of synthesis	Time	Temp (in ˚C)	Emission wavelength	Size	Ref
1	PEG400, with EuCl3 · 6H2O	Microwave synthesis	1-6 hrs	150 - 200	615 nm	3-5 nm	(28)
2	pulp-free lemon juice in	Solvothermal	10 hrs	190	631 nm	~4.6 nm	(29)

	ethanol						
3	Citric acid, urea in formamide	solvothermal	12 hrs	180	630 nm	2-3 nm	(30)
4	Ru-Aphen and Citric acid	Hydrothermal	3hrs	185	606-616nm	3-6 nm	(31)
5	Dicyandiamide, o-paraphenylene diamine and sulfuric acid	Hydrothermal	10 hrs	200	630nm, 680 nm	~5.7 1 nm	(32)

SI 2: Fluorescence intensity of CNPs in different solvents

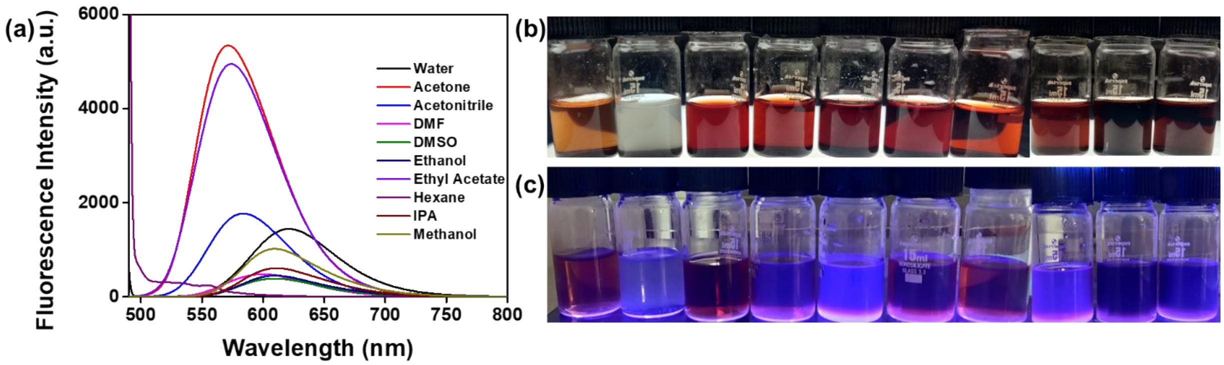

SI 2(a) Emission spectra of CNPs (0.5 mg/mL) dispersed in different solvents (Water, Acetone, Acetonitrile, DMF, DMSO, Ethanol, Ethyl Acetate, Hexane, Isopropinoic Acid, Methanol) **(b) & (c)** Images of CNPs dissolved in different solvents under white light (above) and UV light (below). (From left to right – Water, Hexane, Acetone, Acetonitrile, DMSO, DMF, Ethyl acetate, IPA, ethanol, methanol)

SI 3: Stability check of CNPs in water for seven days

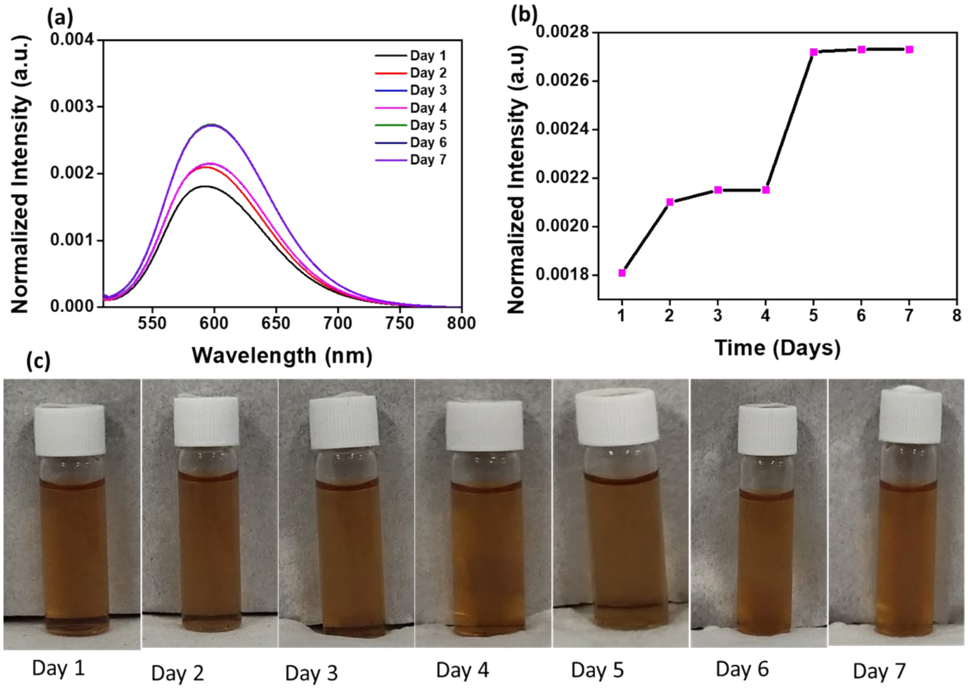

Figure SI 3: Stability studies of CNPs. **(a) and (b)** normalized fluorescence intensity reading taken for seven days,**(c)** Images of 0.5mg/ml CNPs dispersed in water for seven days to check their stability.

SI 4: Fluorescence intensity of CNPs and PPDA

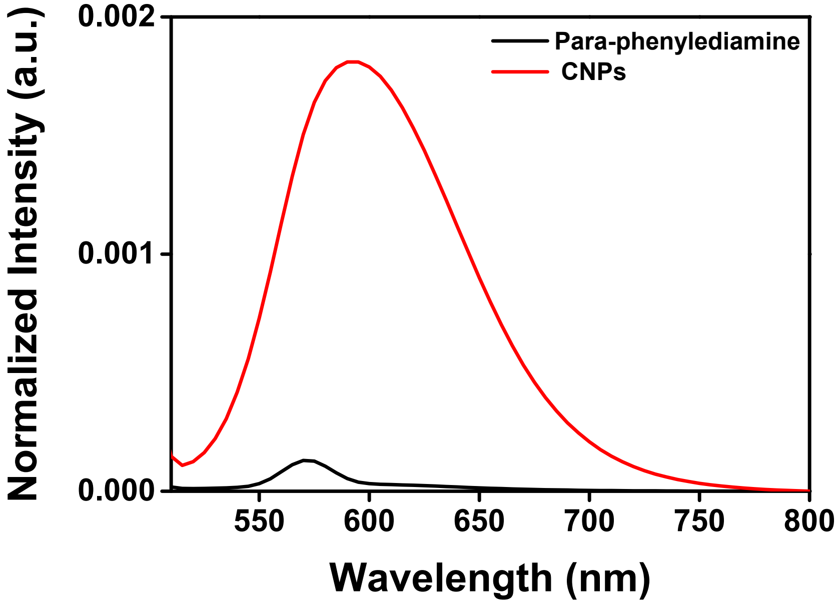

SI 4: Fluorescence intensity comparison of PPDA and CNPs at 480 nm excitation wavelength.

SI 5: MTT assay

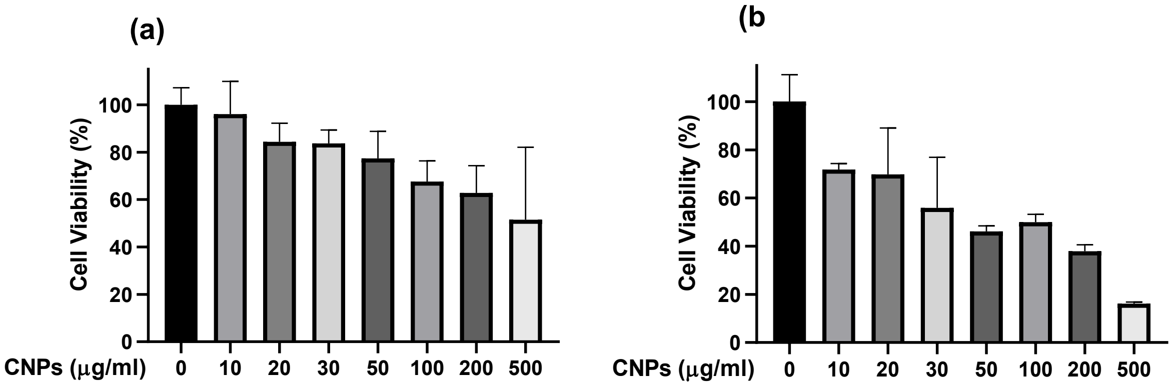

Figure SI 5: Cytocompatibility studies. Cell viability was determined by MTT assay using **(a)** SUM 159 and **(b)** MeFs. The cells were treated with CNPs at 10, 20, 30, 50, 100, 200 and 500 μg/mL for 24 h.

SI 6: 2D Cellular studies of CNPs via confocal microscopy

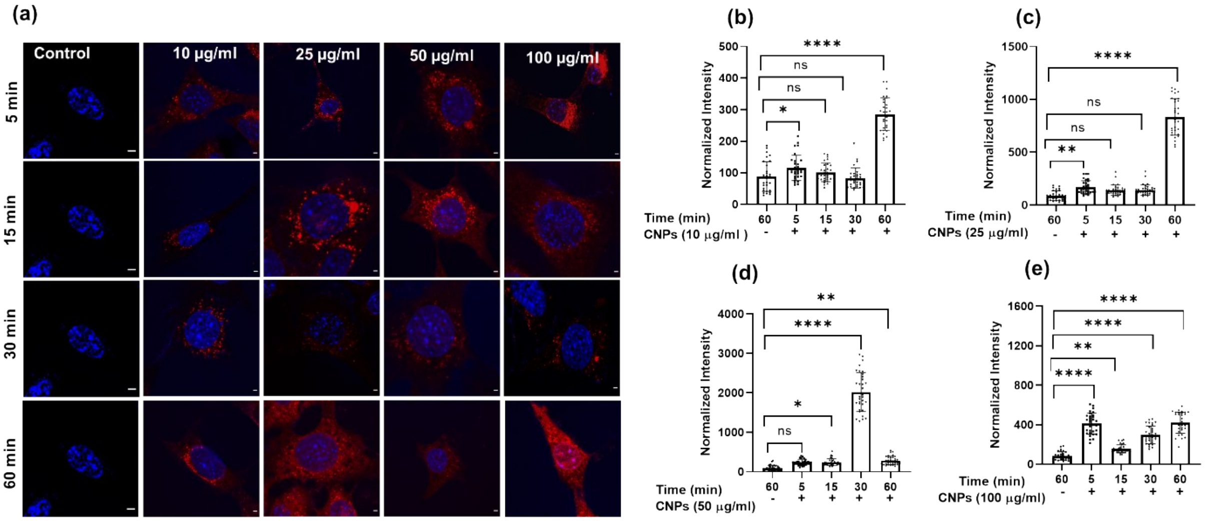

Figure SI 6(i): Concentration dependent cellular uptake studies of CNPs at different time intervals. (a) Confocal images of MeFs incubated with 10, 25, 50 and 100 µg/ml of CNPs respectively. First column in the figure represents untreated MEF cells imaged after 60 min of incubation. Scale bar is 5 µm. (b-e) Quantification of cellular uptake of at 10, 25, 50 and 100 µg/ml of CNPs at 5, 15, 30 and 60 min respectively. **** Indicates statistically significant value of $p < 0.0001$. ** Indicates statistically significant value of $p = 0.002$, * Indicates statistically significant value of $p = 0.03$ and ns indicates non-significant value of p (one-way ordinary ANNOVA).

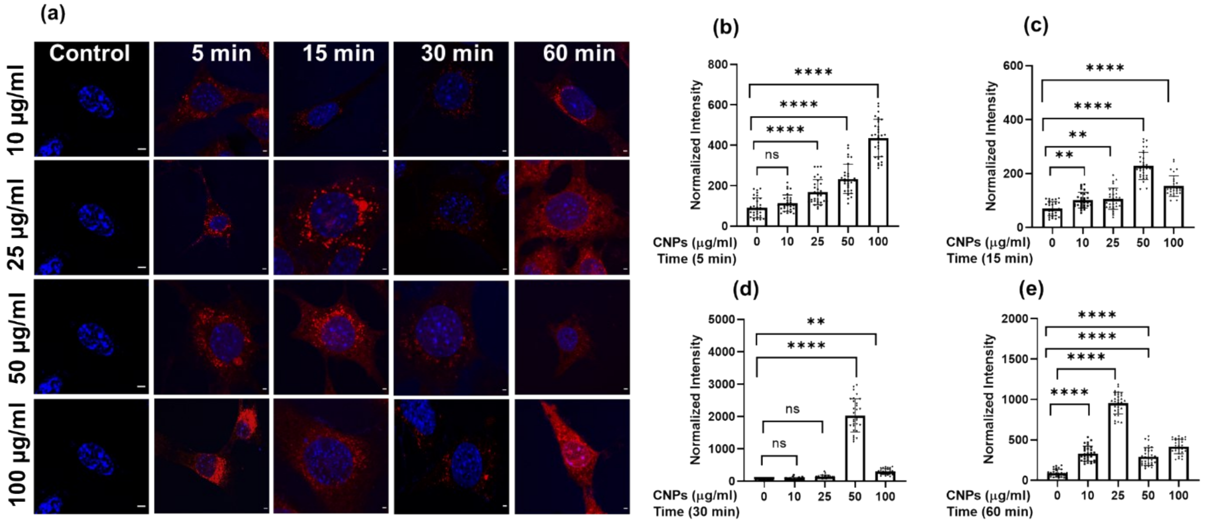

Figure SI 6 (ii): Time dependent cellular uptake studies of CNPs at different time intervals. (a) Confocal images of MEFs incubated with 10, 25, 50 and 100 µg/ml of CNPs respectively. First column in the figure represents untreated MEF cells imaged after 60 min of incubation. Scale bar is 5 µm. (b-e) Quantification of cellular uptake of CNPs at 10, 25, 50 and 100 µg/ml respectively. **** Indicates statistically significant value of $p < 0.0001$, ** Indicates statistically significant value of $p = 0.005$ and ns indicates non-significant value of p (one-way ordinary ANNOVA).
